# Impact of COVID-19 on the Mental Health of Healthcare Workers and Job Loss From a Gender Perspective in India: A Systematic Review and Meta-Analysis

**DOI:** 10.7759/cureus.48219

**Published:** 2023-11-03

**Authors:** Ramesh Athe, Rinshu Dwivedi, Kasha Singh, Sabiha Babusab Hulmani, Nikhita Karadi, Chaithanya Boraiah, Sindhu Vasu

**Affiliations:** 1 Data Science and Intelligent Systems, Indian Institute of Information Technology Dharwad, Dharwad, IND; 2 Science and Humanities, Indian Institute of Information Technology Tiruchirappalli, Tiruchirappalli, IND; 3 Computer Science and Engineering, Indian Institute of Information Technology Dharwad, Dharwad, IND

**Keywords:** healthcare workers, mental health, unemployment, india, socio-economic, meta-analysis, healthcare, social protection, covid-19

## Abstract

The COVID-19 pandemic caused significant stress and anxiety among the general population and healthcare workers (HCWs) worldwide. India is one of the countries severely impacted by the pandemic. This review explores the gender perspective of mental health conditions among HCWs and job loss during the pandemic in India. Electronic databases (PubMed, Scopus, and Web of Science) were searched for articles published till March 2021. Studies that reported the prevalence of depression, anxiety, stress, and worry among HCWs in India during the pandemic and job loss in both males and females due to COVID-19 were included. We used a random-effects model to estimate pooled prevalence rates with 95% CIs. We assessed heterogeneity using the I^2^ statistic. The meta-analysis included 11 studies; the pooled prevalence of depression, anxiety, stress, and worry among HCWs was 34.9% (95%CI 27.33, 42.47), 35.4% (95%CI 24.46, 46.33), 32.9% (95%CI 25.43, 40.37), and 42.87% (95%CI 25.83, 59.91), respectively. The pooled prevalence of job loss due to COVID-19 was 16.6% (95%CI 8.34, 19.66). We employed meta-regression and Egger’s regression for publication bias. The meta-analysis findings suggest that the prevalence of depression, anxiety, stress, and worry among HCWs in India during COVID-19 was high. Furthermore, job loss due to COVID-19 has also been prevalent in India. These findings emphasize the need for mental health support for HCWs and those who have lost their jobs during the pandemic. It is essential to prioritize mental health and job creation policies in India to support individuals affected by COVID-19.

## Introduction and background

Global health, economics, and communities have suffered significantly as a result of the coronavirus disease 2019 (COVID-19) pandemic [1]. The pandemic created substantial difficulties in India, as in many other nations, including higher levels of stress and anxiety among healthcare workers (HCWs) and widespread job loss among both men and women [2,3]. In order to develop evidence-based policies and initiatives to improve the wellbeing of HCWs and vulnerable people, it is imperative to comprehend the pandemic's effects on these two crucial sectors [3].

Doctors, nurses, and other front-line HCWs have been at the vanguard of the COVID-19 response, putting their own health and wellbeing at risk to care for people who have been infected. HCWs throughout the world, especially in India, are suffering from the pandemic's unprecedented demands, including increased workload, shortage of personal protective equipment (PPE), fear of infection, and seeing patients suffer. HCWs' stress and anxiety levels not only affect their personal health but may also have an impact on the level of patient care they deliver [2,3].

On the other hand, a significant economic impact of the pandemic has been job loss, particularly in nations with sizable informal economies like India. Due to extensive disruptions in industries, supply chains, and service sectors, both men and women have experienced severe employment losses, particularly those in low-income and marginalized communities. Due to their over-representation in informal and vulnerable work, as well as their role as care and household managers, women have been disproportionately affected by job loss, which has raised concerns about how it would affect them on the basis of gender [4,5].

This meta-analysis aims to synthesize the existing literature and provide a thorough overview of the existing evidence to comprehend the effect of COVID-19 on the level of stress and anxiety among HCWs and the resultant job loss among men and women in India.

The present review has two objectives: first, it studies how the COVID-19 pandemic has affected stress and anxiety levels among Indian HCWs, and second, it examines how the pandemic has affected job losses in both men and women in India. With the help of this meta-analysis, we hope to learn more about how common stress and anxiety are among HCWs, what triggers them, and how well interventions work to treat these psychological side effects. Additionally, we observe how the pandemic has affected employment loss differently among men and women in India, as well as the causes of job loss and the efficacy of policy initiatives to lessen the impact on vulnerable populations.

The findings from the present meta-analysis anticipate an addition to the existing body of knowledge on the COVID-19 pandemic and its effects in India by highlighting the repercussions in academics, policymaking, and HCWs. The results can be referred to for evidence-based policy formulation and probable interventions for improving the mental health and overall wellbeing of HCWs. Also, the results can be utilized for supporting vulnerable populations through various policy measures that were adversely affected by job loss in the midst of the continuing epidemic and any such future crises.

## Review

Methodology

In order to provide an overview of the level of depression, anxiety, stress, and worry among HCWs and job loss among men and women in India due to COVID-19, the steps of this process were conducted according to Preferred Reporting Items for Systematic reviews and Meta-Analysis (PRISMA) guidelines for meta-analysis [6-8].

Literature Search

The search was conducted in multiple databases such as PubMed, Embase, Scopus, and Google Scholar along with secondary references of included studies [8-10]. The search included keywords related to COVID-19, stress, healthcare workers, job loss, and men and women in India and articles published between November 1, 2019, and January 31, 2023. Both Medical Subject Headings (MeSH) terms and free-text terms were used. The following search terms were used to identify relevant articles.

Mental Health: (("COVID-19" OR "Coronavirus" OR "SARS-CoV-2") AND ("Stress" OR "Mental Health" OR "Psychological Distress") AND ("Healthcare Workers" OR "Medical Personnel" OR "Hospital Personnel") AND "India")

Employment: (("COVID-19" OR "Coronavirus" OR "SARS-CoV-2") AND ("Job Loss" OR "Unemployment" OR "Employment") AND ("Men" OR "Women" OR "Gender") AND "India")

Inclusion Criteria

The search period used was from November 1, 2019, to January 31, 2023. To reduce publication bias, both published and unpublished studies including gray literature like dissertations and conference proceedings were included. A mixed design was applied for the present study where all prospective randomized controlled trials (RCTs) emphasizing and evaluating the impact of COVID-19 on the mental health of the HCWs and job loss among men and women were included. The criteria of inclusion were based on depression, anxiety, and stress along with trials that compared intervention groups with placebo control groups. Only trials that examined the following were included: mental health, COVID-19, HCWs, job loss, gender, and India. Inclusion criteria in the various categories are given below.

Population: For the analysis of depression, anxiety, and stress, studies that included HCWs such as doctors, nurses, and other healthcare professionals, were included. For the analysis of job loss, studies involving men and women from a variety of occupational backgrounds were incorporated.

Intervention: The studies encompassed cross-sectional, cohort, case-control, and RCTs, among other comparators.

Outcome: Studies that evaluated depression, anxiety, and stress levels in HCWs using quantitative techniques such as questionnaires, self-report scales, or objective measurements were included. Also, studies that used quantitative methods/approaches to quantify and measure job loss among men and women, such as self-reported job loss, employment status, or objective measurements of job loss were also included in the present study.

Exclusion Criteria

Studies lacking pertinent outcome measures or which did not track depression, anxiety, stress, or job loss among HCWs or among men and women were disqualified. Literature reviews, case studies, and qualitative research were disregarded because the meta-analysis only considered quantitative research. Studies published in languages other than English were excluded [8-10].

Data Extraction and Quality Assessment

We have administered the data retrieval on a piloted standardized form in Microsoft Excel (Microsoft Corporation, Redmond, Washington, United States) and reviewed it. The title and abstract of the studies in web searches were identified and reviewed, and irrelevant studies were excluded. Furthermore, the full text was retrieved for the included studies. Data extracted consisted of the interventions used to address depression, stress, anxiety, and mental health among HCWs and job loss, and unemployment from the gender perspective during the pandemic in India. We also retrieved data related to the authors, year of publication, country, pandemic, design and method, participants, mental health issues, and in context to unemployment or job loss [8-10]. The search, data extraction, and quality assessment were completed independently by two content experts according to the inclusion criteria and confirmed using recommended criteria for RCTs and quantitative assessments. The Joanna Briggs Institute critical appraisal checklist for studies reporting prevalence data was used to determine and control the articles’ quality. This instrument was used for the quality assessment of articles, which contains questions responded to via three options: yes, no, and not applicable [11]. The instrument aimed at evaluating the methodological quality of articles and determining errors in studies, designs, and data analysis. The results for the quality of studies indicated that all included studies had been qualified as per the quality standards for the final analysis.

Statistical Analysis

The major focus of the study was to explore the mental health conditions with reference to depression, anxiety, and level of stress among HCWs and job loss from the gender perspective during the COVID-19 pandemic in India. An estimation of the overall effect size regarding the impact of COVID-19 on the level of depression, anxiety, and induced stress among Indian HCWs was identified using a random-effects meta-analysis methodology due to the presence of heterogeneity (τ2 > 0). The effect size, which is the difference between depression, anxiety, and stress scores of HCWs before and during the COVID-19 pandemic, is referred to as the standardized mean differences (SMD) and was calculated for each included trial.

Further, for assessing the impact of COVID-19 on job loss among men and women in India, a fixed-effects meta-analysis model was applied to estimate the overall effect size with a 95% confidence interval (CI). The effect sizes were measured using odds ratios (OR) between the number of job losses among men and women before and during the COVID-19 pandemic. Once an effect size was estimated for each trial, the overall effect of these results was assessed by Cochrane's Q statistic, which measures consistency among studies. The Q test was computed under the assumption of homogeneity among the effect sizes and the statistic follows the Chi-square distribution with k-1 degrees of freedom (DFS), where k is the number of studies. Another method for quantifying the heterogeneity among the studies in a meta-analysis consisted of estimating the variance (τ2) between studies. The parameter I2 quantified the percentage of total variation in study estimates due to heterogeneity rather than sampling error. The overall SMD and the OR of these results were measured for sampling error (homogeneous; τ2 = 0).

The heterogeneity of results was depicted in the form of a forest plot, which typically represents a blob in the middle of the 95%CI that characterizes the OR estimates. The forest plot presents the graphical representation of the results. The horizontal line in the middle of the plot represents the overall effect size, and the pooled or combined result of the SMD and OR in effect size is denoted by diamonds on the plot representing 95%CI for each study or subgroup for the combined data. The size of the diamond reflects the weight assigned to each study, and the horizontal line through the diamond represents the point estimate of the prevalence rate. The vertical line represents the null effect line, indicating no difference between the experimental and control groups. The forest plot also shows Cochrane's Q statistic, τ2, df, I2, Z, and ρ value. The I2 statistic and funnel plots were administered to measure the publication bias and presence of heterogeneity between the included studies respectively [8-10].

An I2 > 50% indicates a significant heterogeneity between the trials. The meta-regression analysis was performed to detect the source of heterogeneity (I2>50%) of depression, anxiety, stress, and unemployment. Publication bias was assessed with the funnel plot and Egger regression test. Publication bias is present when studies with minor or non-significant results are less likely to be published than studies with large and significant results. Egger's regression is a statistical approach used in meta-analysis to evaluate the presence of publication bias. A modified version of the traditional regression model called Egger's regression takes into consideration the likelihood of publication bias or small study effects in the meta-analysis. If there is evidence of heterogeneity, a meta-regression approach is used to test the heterogeneity by relating study characteristics. The major confounders were identified, followed by a meta-analysis to estimate the net pooled effect size, after standardizing the effect of confounding variables. To investigate the link between study-level covariates and the effect size estimates gleaned from individual research, a meta-regression approach was used. Statistical analyses were performed with Review Manager (RevMan) software version 5.3 (The Cochrane Collaboration, London, United Kingdom) and Stata Statistical Software: Release 14 (2015; StataCorp LLC, College Station, Texas, United States).

Results

Search Results

**Figure 1:**
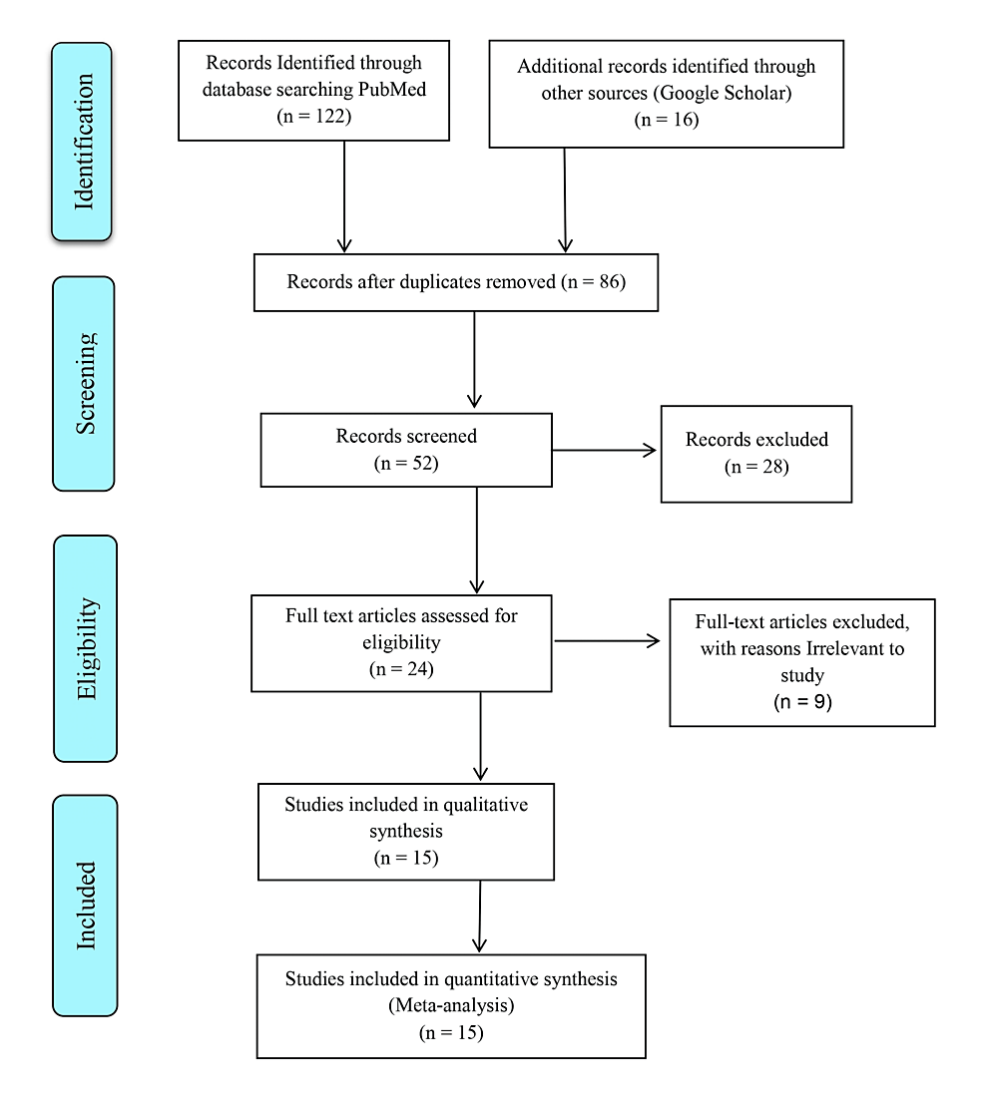
PRISMA flow chart of the included studies PRISMA: Preferred Reporting Items for Systematic reviews and Meta-Analysis

A total of 138 (N=138) articles were identified, of which 86 were excluded as they were not relevant to the purpose of the current analysis. Further, with the screening of the titles, 52 potentially relevant articles were selected for full-text evaluation. Finally, 15 potentially relevant studies [12-26] were included for meta-analysis after employing inclusion and exclusion criteria as depicted in Figure 1 and summary statistics tabulated in Table 1.

**Table 1:** Summary of included studies assessing the effect of depression, anxiety, stress, and worry in the COVID-19 pandemic COVID-19: coronavirus disease 2019

Author, Year	Study Design	Sample size			Prevalence %			
		Total	Male	Female	Depression	Anxiety	Stress	Worry
Chatterjee et al., 2020 [12]	Cross-sectional, observational study	152	119	33	34.9	39.5	32.9	-
George et al., 2020 [13]	Mixed methods	64	24	40	-	73.4	62.5	81.2
Grover et al., 2020 [14]	Cross-sectional survey	144	66	78	53	52	14	-
Gupta et al., 2021 [15]	Prospective study	1124	718	406	31.4	37.2	-	-
Gupta et al., 2020 [16]	Cross sectional	749	556	193	28.2	35.2	-	-
Nanjundswamy et al., 2020 [17]	Survey	106	-	-	-	35	-	73
Pandey et al., 2020 [18]	Cross-sectional survey	83	36	47	7.3	9.8	-	44.5
Saraswathi et al., 2020 [19]	Prospective longitudinal	217	78	139	35.5	33.2	24.9	-
Sharma at al., 2020 [20]	Questionnaire-based observational cross-sectional	200	-	-	72	85	82	-
Suryavanshi at al., 2020 [21]	Structured online survey	197	96	101	22	29	-	-
Podder et al., 2020 [22]	Web‐based cross‐sectional study	384	213	171	-	-	85.9	-

Effect of COVID-19 on Mental Health of HCWs

**Figure 2:**
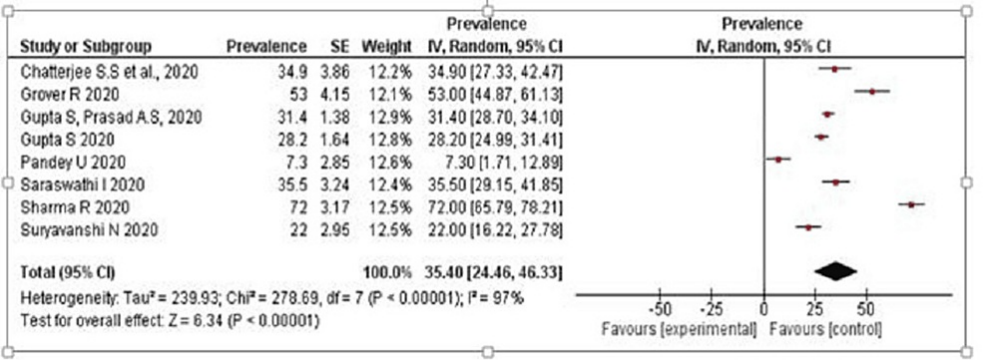
Prevalence rate of depression among healthcare workers References: [12,14-16,18-21]

Depression: From the forest plot (Figure 2), the results indicate that the prevalence rate of depression among HCWs in India during the COVID-19 pandemic ranges from 7.3% to 72%, with a pooled estimate of 35.4% (95%CI: 24.46-46.33). The random effects model was used for the analysis, and the heterogeneity statistics showed a high degree of heterogeneity (Tau 239.93; Chi=278.69; df= 7; p< 0.00001; I2=97%) that indicates variation in prevalence estimates across the studies.

**Figure 3:**
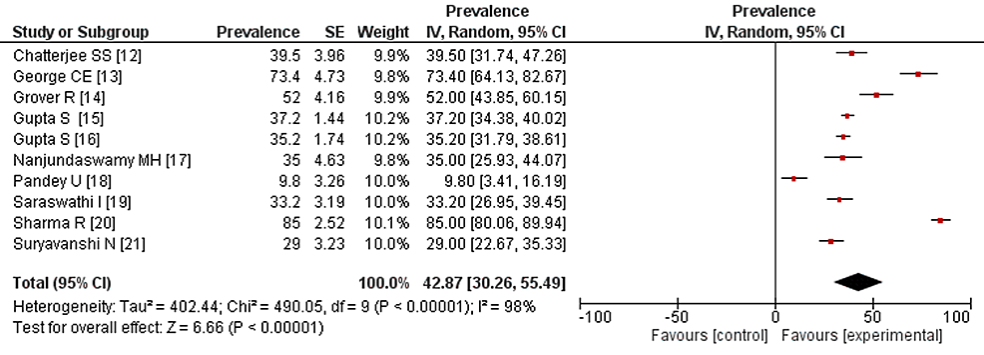
Prevalence rate of anxiety among healthcare workers. References: [12-21]

Anxiety: The results (Figure 3) indicate that the prevalence rate of anxiety among HCWs in India during the COVID-19 pandemic ranges from 9.8% to 73.4%, with a pooled estimate of 42.87% (95%CI: 30.26-55.49). The random effects model was used for the analysis, and statistically significant heterogeneity was observed (Tau 402.44; Chi 490.05, df= 9; p < 0.00001; and I2 =97%) across the studies.

**Figure 4:**
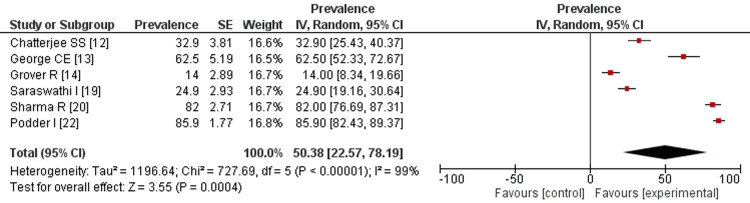
Prevalence rate of stress among healthcare workers. References: [12-14,19,20,22]

**Figure 5:**
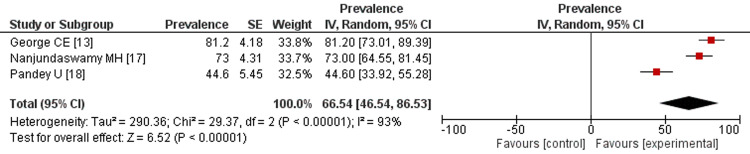
Prevalence rate of worry among healthcare workers. References: [13,17,18]

Stress: Figure 4 represents the prevalence rate of stress among healthcare workers in India reported from the included studies. The meta-analysis of these studies showed that the overall prevalence rate of stress among HCWs in India is 50.38% (95%CI: 22.57-78.19), with significant heterogeneity (Tau = 1196.64, Chi = 727.69, df = 5, P < 0.00001). The prevalence rate of worry among HCWs in India is estimated to be 66.54%, (95% CI: 46.54-86.53), which is depicted in Figure 5. Also, significant heterogeneity has been observed between the studies (Tau=290.36, Chi=29.37, df=2, P<0.00001, I-squared=93%).

Effect of COVID-19 on Job Loss in India From a Gender Perspective

**Figure 6:**
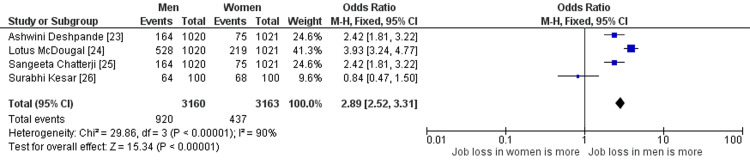
Unemployment among citizens of India due to COVID-19 References: [23-26] COVID-19: coronavirus disease 2019

The analysis included four studies [23-26] on this with a total of 3160 participants, out of which 1020 were men and 1021 were women. The results show that the odds of job loss were significantly higher among men as compared to women, with an OR of 2.89 (95%CI 2.52-3.31), which is depicted in Figure 6. It indicates that men were at more than two times higher risk as compared to women counterparts to lose their jobs due to COVID-19 in India.

The heterogeneity among the studies was reported to be significant (Chi 29.86, df = 3, P < 0.00001; I2= 90%), indicating that the studies included in the analysis varied considerably in their results. However, the overall effect was significant (Z=15.34, P < 0.00001), indicating that the observed difference in job loss between men and women is likely to be real and not due to a chance factor.

Findings From the Meta-Regression

Meta-regression analysis was performed to detect the sources of heterogeneity [8-10,27,28]. We took three different models based on: (i) depression, (ii) anxiety, and (iii) stress. The results indicate a notable association between the logarithmic event rate and the covariates in respective models.

**Figure 7:**
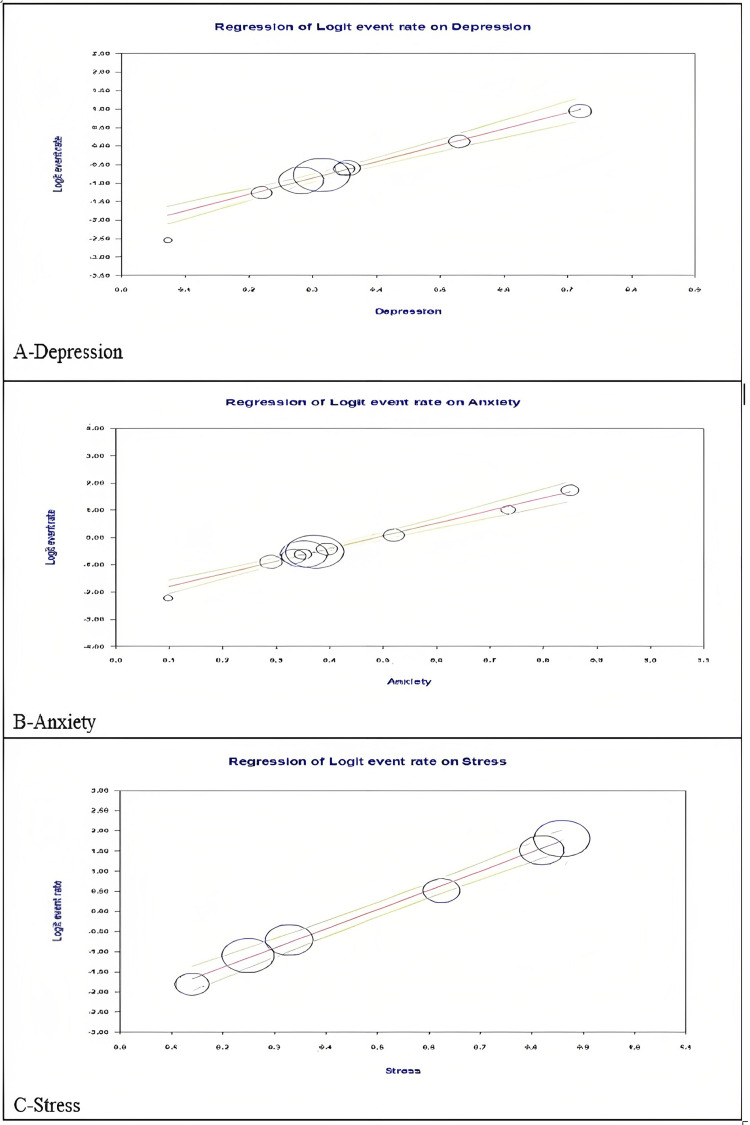
Scatter plot depiction of the prevalence rate of (A) depression (B) anxiety, and (C) stress among healthcare workers.

Depression: By examining the distribution and pattern of the data points in Figure 7A, we can assess the relationship between the predictor and the outcome variable. The graphical representation indicates trends from lower depression levels to higher depression levels among HCWs during COVID-19. We can see an increase in the logarithmic values of event rate (prevalence). The tau^2 value indicates the estimated amount of between-study variability in the outcome variable (depression) that cannot be explained by the predictor variable (logit event rate).

Anxiety: The plot in Figure 7B represents the results of the meta-regression analysis related to anxiety. It displays the relationship between the predictor variable (logarithm of event rate) and the level of anxiety. The graphical representation indicates trends from lower anxiety levels to higher anxiety levels among HCWs during the COVID-19 pandemic. We can see an increase in the logarithmic values of event rate (prevalence).

Stress: The scatter plot in Figure 7C provides insights into heterogeneity and variability among the studies included in the meta-regression analysis. The graphical representation indicates trends from lower levels of stress to higher levels of stress among HCWs during the COVID-19 pandemic. We can witness an increase in the logarithmic values of the event rate. HCWs often face emotionally challenging situations, which include witnessing severe illness, death, and suffering of patients. The constant exposure to such distressing situations can take a toll on their emotional well-being, leading to increased stress and burnout.

Publication Bias and Egger’s Regression

**Figure 8:**
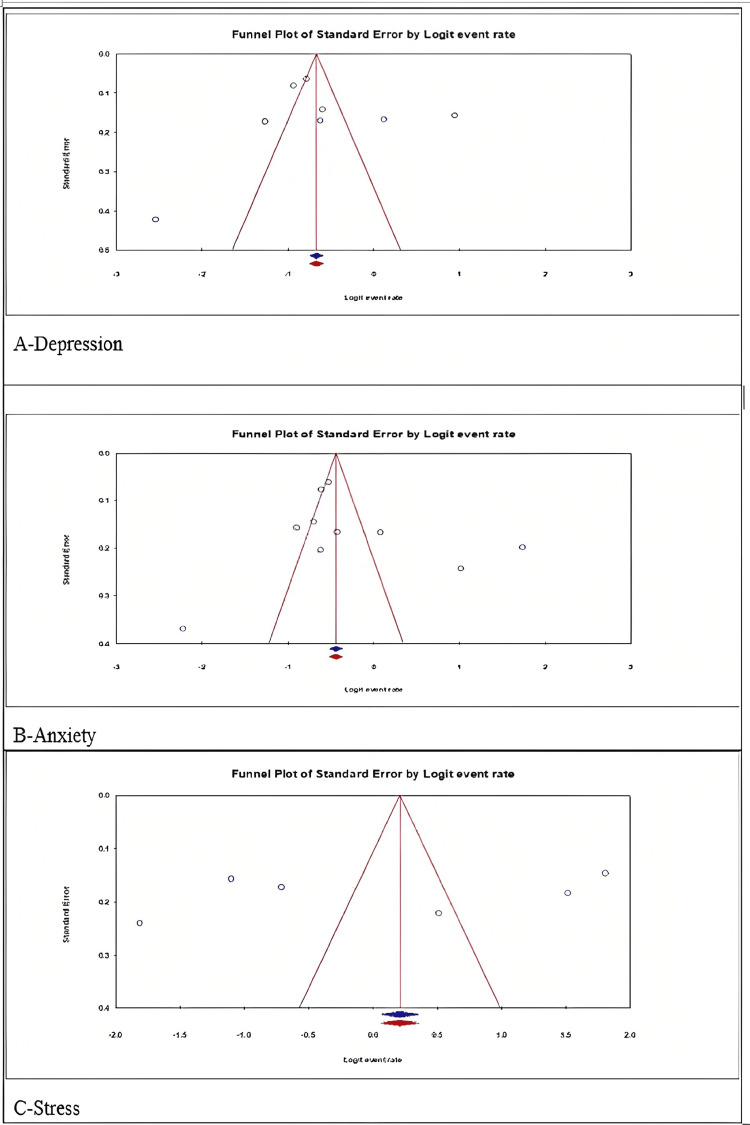
Funnel plot depiction of the prevalence rate of (A) depression (B) anxiety, and (C) stress among healthcare workers.

The funnel plot (Figure 8) was symmetrical, indicating the probable absence of publication bias which was confirmed using Egger’s weighted regression method [8,27,28]. The inverse of the sample size of each study that was included in the meta-analysis is regressed against the standard error of the effect size estimate in Egger's regression. Egger’s regression has confirmed that there is no publication bias (Depression, p=0.6977; Anxiety, p=0.4467; and Stress, p=0.6976). Detailed analysis is mentioned in the Appendices. 

Discussion

The COVID-19 pandemic became a serious problem of mental anguish among HCWs [4,5]. HCWs suffered from stress, depression, anxiety, and insomnia during the COVID-19 pandemic [29]. Gender, age, place of work, profession, and department of work were notably associated with increased anxiety, stress, and insomnia among HCWs during the pandemic [30]. Extended working hours, emergency calls, quarantine, and separation from friends and family due to professional commitments put HCWs under extreme stress, anxiety, and frustration. They were also worried about transferring the infection to their loved ones and others [15,19,20].

The study-specific prevalence rates of depression ranged from 7.3% to 85%, and the sample sizes of the studies ranged from 29% to 73.4%. The largest weight in the meta-analysis was given to the study by Sharma et al., which reported a prevalence rate of 72% with a sample size of 22 [18]. The smallest weight was given to the study by Nanjundaswamy et al., which estimated a prevalence rate of 35 and a sample size of 4 [21]. The study by Podder et al. reported the highest prevalence rate of anxiety (85.9%) [22], followed by the study of George et al. with a prevalence rate of 62.5% [20]. The study by Sharma et al. reported the lowest prevalence rate (2.93%) [18]. By doing the meta-analysis and creating forest and funnel plots, we were able to verify that HCWs faced a lot of stress during the pandemic.

The impact of COVID-19 on job loss among men and women in India revealed that both genders were affected by the pandemic's economic downturn [31,32]. However, men experienced a higher proportion of job loss as compared to women. This finding could be attributed to pre-existing gender disparities in the labor market, such as lower participation rates and unequal pay. The meta-analysis's limitations comprised the heterogeneity of the included studies' methods and data sources, which could have affected the comparability of the results. Additionally, the analysis was based on the existing literature, which may not fully capture the extent of the pandemic's impact on job loss in India from the gender perspective.

## Conclusions

The COVID-19 pandemic significantly influenced the mental health of HCWs in India, leading to an increase in depression, anxiety, and induced stress levels. The findings of this systematic review and meta-analysis suggest an urgent need for targeted interventions to support the mental health of HCWs during and beyond the pandemic. Such interventions should include psychological support, stress management, and access to mental health services. It is essential for policymakers and healthcare organizations to prioritize the wellbeing of HCWs to ensure that they can continue to provide high-quality care to patients while protecting their own mental health. Further research should explore effective interventions to reduce the burden of depression, anxiety, and stress among HCWs in India during the COVID-19 pandemic.

Regarding COVID-19's effects on employment loss among men and women in India, women disproportionately realized the economic burden of the pandemic. The study's findings highlight the necessity for policies and programs that take into account gender when addressing the pandemic's diverse effects on various job outcomes. Further study is also required in order to establish successful methods to promote gender equality in the labor market and to point out the underlying causes for gender differences found through the analysis. India can lessen the impact of the pandemic on employment and support a more equitable and sustained economic recovery by adopting a more in-depth study on why these differences exist in the Indian labor market. This can be crucial from the policy perspective by adopting a gender-inclusive approach to both policy and action.

## Appendices

**Table 2:** Meta-regression and Egger’s regression analysis of depression, anxiety, and stress among included studies.

Meta Regression Analysis							
Covariate	n	Co-ordinates	Coefficient	Standard error	95% CI		p-value
					Lower	Upper	
Depression	8	Intercept	-2.192	0.123	-2.435	-1.950	0.000
		Depression	4.427	0.339	3.762	5.092	0.000
Anxiety	10	Intercept	-2.261	0.132	-2.520	-2.001	0.000
		Anxiety	4.647	0.323	4.013	5.280	0.000
Stress	6	Intercept	-2.345	0.156	-2.652	-2.038	0.000
		Stress	4.774	0.259	4.266	5.282	0.000
Egger’s Regression Analysis							
Depression	8	Intercept	1.67360	4.10658	-8.37483	11.72203	0.697
Anxiety	10	Intercept	2.61283	3.18431	-4.73019	9.95586	0.435
Stress	6	Intercept	-15.98374	20.86297	-73.90863	41.94115	0.486
